# Patterns of Transmission and Sources of Infection in Outbreaks of Human Toxoplasmosis

**DOI:** 10.3201/eid2512.181565

**Published:** 2019-12

**Authors:** Fernanda Pinto-Ferreira, Eloiza Teles Caldart, Aline Kuhn Sbruzzi Pasquali, Regina Mitsuka-Breganó, Roberta Lemos Freire, Italmar Teodorico Navarro

**Affiliations:** State University of Londrina, Londrina, Brazil (F. Pinto-Ferreira, E.T. Caldart, R. Mitsuka-Breganó, R.L. Freire, I.T. Navarro);; Unoesc, Santa Catarina, Brazil (A.K.S. Pasquali);

**Keywords:** *Toxoplasma gondii*, oocysts, water, vegetables, human, outbreak, toxoplasmosis, parasites

## Abstract

We report on apparent temporal progression of probable sources of infection and transmission routes for global human toxoplasmosis outbreaks as described in published articles. We searched the Scientific Electronic Library Online, Web of Science, PubMed, and Scopus databases for articles on *Toxoplasma*, toxoplasmosis, and outbreaks. We found that transmission routes for *Toxoplasma gondii* varied by decade. In the 1960s and 1990s, toxoplasmosis outbreaks mainly occurred through ingestion of cysts in meat and meat derivatives; in the 1980s, through milk contaminated with tachyzoites; in 2000, due to the presence of oocysts in water, sand, and soil; and in 2010, due to oocysts in raw fruits and vegetables. Our study suggests a possible change in the epidemiology of reported toxoplasmosis outbreaks. Because of this change, we suggest that greater attention be paid to the disinfection of vegetables, as well as to the quality of water used for drinking and irrigation.

Toxoplasmosis is a zoonotic disease caused by the protozoan *Toxoplasma gondii* of the phylum Apicomplexa, an obligate intracellular parasite with a worldwide distribution that infects mammals and birds (*1*). Warm-blooded animals serve as intermediate hosts for *T. gondii*, but felids are its only definitive host and shed oocysts that result in environmental contamination (*2*).

Because of high exposure to *T. gondii* around the world, humans have a high serologic prevalence, which varies between 10.0% and 97.4% in the adult population. However, cases of clinical disease are less frequent (*3*). Environmental conditions, cultural and eating habits, and fauna are factors in the variability and prevalence of toxoplasmosis in different geographic areas (*4*). Transmission mainly occurs through the ingestion of water, vegetables, or soil contaminated with oocysts (sporozoites) or raw or undercooked meat containing viable tissue cysts (bradyzoites), characterizing this disease as a foodborne zoonosis (*3*).

Better understanding of the patterns of occurrence of human toxoplasmosis outbreaks could lead to more effective and targeted prevention and control measures. We report a possible temporal progression of the probable sources of infection and transmission routes described in articles on human toxoplasmosis outbreaks throughout the world from the 1960s through March 2018.

## Materials and Methods

We performed a systematic review by searching the Scientific Electronic Library Online (Scielo), Web of Science, PubMed, and Scopus databases by using the keywords “*Toxoplasma* AND outbreak OR toxoplasmosis AND outbreak.” During February–March 2018, we collected data on published toxoplasmosis outbreaks in humans since 1967, when the first relevant article on human infection was published (*5*). 

We reviewed published articles to look for changes in the pattern of transmission routes and sources of infection for toxoplasmosis outbreaks in humans around the world. We included articles with at least the abstract in English or Portuguese. We excluded articles on outbreaks of toxoplasmosis in nonhuman species and studies without information about the transmission route.

For each outbreak report, we extracted the year, country of outbreak occurrence, probable source of transmission, number of affected persons, and the affected group. By reviewing the probable source of infection and transmission route defined by the authors of the selected papers, we inferred the parasitic form involved in each case or outbreak report. We used Mendeley (Elsevier, https://www.mendeley.com) software to organize, exclude, and select references. We used Epi Info 3.5.4.4 (*6*) software to tabulate variables obtained from information extracted from the selected articles. 

We performed statistical analyses by using χ^2^ or Fisher exact tests in R 3.4.1 (http://www.R-project.org) and performed multiple correspondence with the FactoMineR package (http://factominer.free.fr). We chose this technique because it does not rely on statistical tests and provides visualization of the most relevant relationships in a large set of variables (*7*). It also helps visualize the multivariate relationship between categories of different variables; the geometric proximity of variables in the graph suggests their possible association.

## Results

**Figure 1:**
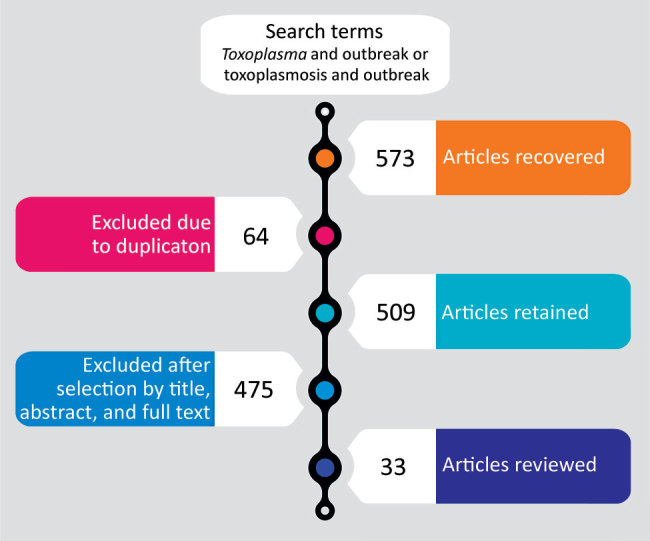
Selection process for articles for systematic review of reports of outbreaks of human toxoplasmosis throughout the world during 1967–March 2018.

We found a total of 573 articles: 10 in Scielo, 224 in Web of Science, 83 in PubMed, and 256 in Scopus. We excluded articles that did not contain the likely route of transmission, as well as duplicate or incomplete articles, such as those missing titles, authors, or abstracts. We also excluded articles we could not access because they were not available on the Internet or in other sources. For analysis, we selected 33 articles covering 34 reports of outbreaks of acute toxoplasmosis (Figure 1; Appendix Table).

**Figure 2:**
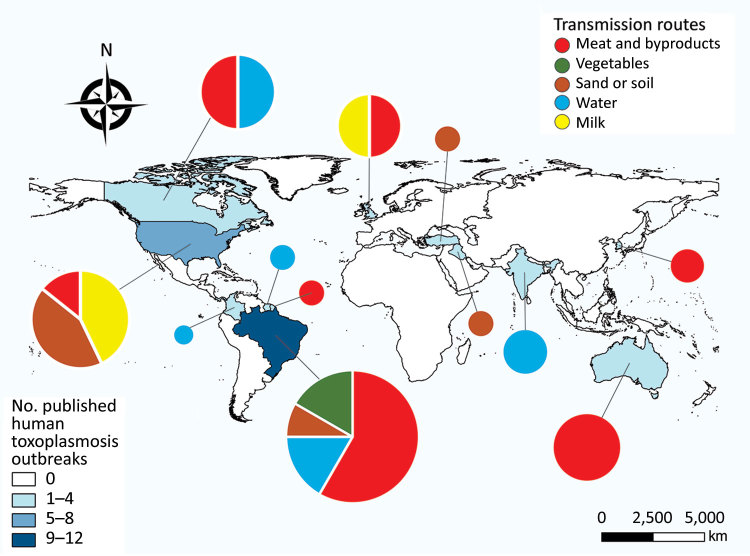
Geographic distribution of 34 outbreaks of human toxoplasmosis worldwide as cited in reports published during 1967–March 2018. Probable and known transmission routes are indicated by color. Circle size corresponds to the number of outbreaks.

We plotted the geographic distribution of the selected outbreaks on a map (Figure 2). The highest concentration of reported outbreaks, 25/34 (73.5%), occurred in the Americas; Brazil had 35.3% (12/34) of the published outbreaks.

The incidence of cyst-related outbreaks from contaminated meat and its derivatives was 47.1% (16/34), and oocysts were implicated in 44.1% (15/34) of the outbreaks. Transmission through the intake of oocysts in water occurred with a frequency of 20.6% (7/34), through contact with sand and soil with a frequency of 17.6% (6/34), and through consumption of vegetables with a frequency of 5.9% (2/34). Tachyzoites in raw milk caused 8.8% (3/34) of outbreaks. Approximately 1,416 persons were affected in the 15 outbreaks of toxoplasmosis from oocysts (sporozoites), 290 in the 16 outbreaks from tissue cysts (bradyzoites), and 15 in the 3 outbreaks from tachyzoites.

**Figure 3:**
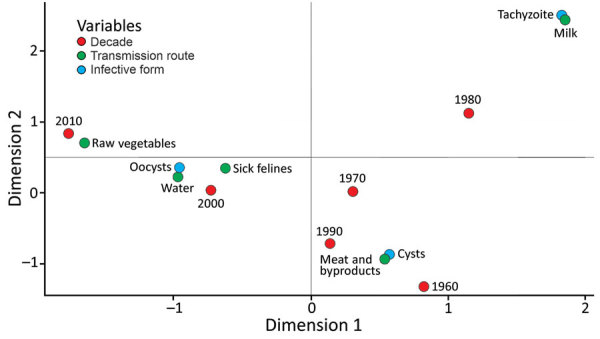
Multiple correspondence analysis of the variables extracted from articles published on human toxoplasmosis outbreaks during 1967–March 2018. The multiple correspondence technique helps to visualize the multivariate relation between the categories of different variables. Proximity of the variables in the graph suggests a possible association between them.

We did not observe a statistical significance in the variables extracted from the articles. Our multiple correspondence analysis shows outbreaks mainly occurred through the ingestion of cysts in meat and its derivatives in the 1960s and 1990s. In the 1980s, milk contaminated with tachyzoites was the primary transmission route. In 2000, outbreaks were caused by oocysts in water and contact with feline feces. Since 2010, outbreaks related to oocyst intake from raw vegetables have increased (Figure 3).

## Discussion

Our study had several limitations. Many outbreaks are published only in gray literature, such as in nonindexed journals, on government websites, and in conference abstracts, rather than in peer-reviewed journals such as those we searched. Our search was limited to articles with at least the abstract written in Portuguese or English. Reports had long lag time between an outbreak and the publication of the occurrence, ranging from 3–7 years. Transmission routes, which can be measured only by means of epidemiologic investigation (*8*,*9*), were not always defined in the reports. Although our review searched reports from scientific literature worldwide, it demonstrates much of the reality in Brazil, where we saw the most outbreak reports and the highest numbers of affected persons (776/1,721).

The major clones of *T. gondii*, genotypes I, II, and III, described in the literature differ in virulence and epidemiology (*10*–*13*). We did not see a clear domain of any genotype in the United States, even though some have relatively higher frequencies (*12*). In Brazil, the seroprevalence of toxoplasmosis in humans ranges from 21.5% to 97.4% (*14*), with more frequent occurrences of atypical genotypes, which might explain reports of the more severe form of the disease (*15*) and the larger numbers of affected persons from this country. In fact, the 2 largest outbreaks of human toxoplasmosis we saw occurred in Brazil. In 2001, an outbreak in Santa Isabel do Ivaí in Paraná State involved >400 persons and was attributed to contamination of the municipal water supply network (*16*). Another outbreak occurred in Santa Maria in Rio Grande do Sul State in 2018 and affected >900 persons; the cause has yet to be determined (*17*).

Oocysts and cysts are the most frequent parasitic form of *T. gondii* transmitted to humans (*8*). According to our results, before 1990, cysts consumed in meat were the main biologic form infecting humans. Beef was the suspected vehicle of transmission in 3 outbreaks: 1 in the United States that affected 5 persons, 1 in Brazil that affected 6 persons, and 1 in Brazil that affected 99 (Appendix). However, because cattle have a low ability to form tissue cysts, beef has less epidemiologic value (Appendix).

Human dietary preferences also can facilitate infection by *T. gondii* (*4*), especially in raw or undercooked meat. For example, consumption of kibbe, a traditional dish in the Middle East made from raw lamb meat, was the cause of 5 outbreaks between 1975 and 2006, 2 in Brazil (*18*,*19*) and 1 each in England (*20*), the United States (*21*), and Australia (*22*).

Because tissue cysts are sensitive to heat (*23*), properly cooked meat poses less of a risk for *T. gondii* infection in humans. In the 1990s, the government of Brazil implemented a prevention project for another parasitic infection, teniasis-cysticercosis. The project discouraged consumption of raw or undercooked meats, which likely contributed to the decline in *T. gondii* infections from meat consumption (*24*). 

In addition, the technology of livestock farming has improved management and reduced pathogenic animal infections, making meat safer. In Brazil, to prevent foodborne diseases, the Ministry of Agriculture, Livestock and Supply, through Administrative Rule no. 46, of February 10, 1998, adopted the hazard analysis and critical control point system as a prerequisite to export meat and to improve good production practices (*25*). In addition, manufacturers improved the practice of freezing meats, to either −10°C degrees for 3 days or −20°C degrees for 2 days, which sufficiently inactivates cysts (*26*). After the improvements in the system, the country saw a reduction in seropositivity to *T. gondii* over the intervening years (*8*). 

We noted that consumption of undercooked game meat, such as reindeer, tapir, venison, wild boar, and armadillo, was the cause of *Toxoplasma* infection in several outbreaks globally (*27*–*30*). Studies have demonstrated that the unusual abundance of atypical *T. gondii* strains found in the wild can cause human toxoplasmosis in its most severe form, even in immunocompetent persons (*16*,*27*,*29*–*33*). The increased severity is caused by poor host adaptation to the circulating *T. gondii* zoonotic neotropical strains (*27*). In 2009, Pino et al. (*32*) described severe cardiac involvement in a military man who consumed untreated water during an operation in the jungle. 

In addition, Carme et al. (*27*) reported 16 cases of severe toxoplasmosis in immunocompetent patients hospitalized with nonspecific infectious diseases in French Guiana. Many had severe pulmonary involvement (87.5%), and >1 had visceral alteration. *T. gondii* was isolated from 3 patients and characterized as an atypical genotype. Investigators determined game meat was the source of infection in 31.25% (5/16) of cases, likely through consumption of tissue cysts.

Infection through milk consumption was described in 3 outbreaks during 1975–1988, all of which affected intrafamily groups who consumed raw goat milk (*34*–*36*). Goats are known to secrete tachyzoites in milk (*37*,*38*), and tachyzoites are resistant to processing in fresh cheeses (*39*). Standard measures to prevent *Listeria monocytogenes, Brucella* spp., and *Mycobacterium* spp. contamination in milk also reduce the risk for human *T. gondii* infection. Practices applied in milk production, such as pasteurization and brucellosis and tuberculosis prevention programs, likely have reduced incidence rates of *T. gondii* infection. We saw fewer outbreaks associated with contaminated milk in the 1990s and 2000s.

The outbreak in Santa Isabel do Ivaí (*16*) is notable in the history of toxoplasmosis, not only for the high number of cases but also because *T. gondii* was isolated directly from the transmission source, unfiltered municipal water. After this incident, outbreaks were investigated with more attention to this biologic form, a factor that might explain the increase of detected outbreaks of water origin.

One of the main forms of transmission of toxoplasmosis is the fecal-oral route. Felines, definitive hosts for *T. gondii*, eliminate the oocysts in the environment, where they can remain viable for several months in appropriate conditions and cause infection (*2*). Because cats defecate in soil and sand, contact with these sources is a risk factor for infection. Contact with soil and sand was the transmission route in 17.6% (6/34) of human outbreaks reported; 67.0% of those affected were children or adolescents, probably because children play in these environments and indirectly consume oocysts.

In the past 20 years, consumption of healthy foods, such as vegetables, has increased through efforts to change eating habits and combat obesity (*40*). Vegetables provide micronutrients and fiber, which aid in maintaining body weight (*41*). However, increasing reports of toxoplasmosis have coincided with increased consumption of fruits and vegetables. Toxoplasmosis outbreaks related to vegetables generally occurred because of contamination during the production, including planting, harvesting, transport, and distribution, but also during processing and consumption (*42*). In 2009, an investigation of a cluster of 11 cases of acute toxoplasmosis in a factory with 2,300 employees in São Paulo State, Brazil, revealed vegetable ingestion as the suspected transmission route (*43*). In 2013, the municipality of Ponta de Pedras in Pará State, Brazil, was the scene of an outbreak of toxoplasmosis involving 73 cases with clinical and laboratory findings compatible with the disease. Açaí consumption was identified as the source of the infections. Ponta de Pedras is one of the main producers of açaí in Brazil, but the outbreak occurred during the period when local production of açaí was practically nil. To satisfy the population’s demand for the fruit, açaí vendors acquired the fruit from other locations, where it might have been contaminated with atypical *T. gondii* strains for which the urban population had little immunity (*44*). 

Such events demonstrate the inadequate sanitation in industrial settings and restaurants and lack of quality control of commercial produce common in developing countries. Considering the increased attention given to safety for food of animal origin in recent years, and the concomitant increase in consumption of raw vegetables and fruits, foods contaminated by oocysts could become the main source for toxoplasmosis outbreaks.

When reviewing the number of cases in toxoplasmosis outbreaks, we noted that oocysts were responsible for outbreaks with high case counts, such as those occurring in city districts or entire municipalities. Although reported outbreaks due to oocysts and cysts occurred at similar rates, outbreaks from oocysts affected many more persons (>1,400) than did outbreaks due to cysts (≈290 persons). Contaminated drinking water was responsible for the largest outbreak of toxoplasmosis described (*16*), but water also serves as a contamination route for vegetables and fruits when used in irrigation (*16*,*44*,*45*). Domestic and wild felids are known to have seroprevalence for *T. gondii*, and a single cat can eliminate >100 million oocysts into the environment after primary infection (*46*,*47*). Such shedding can lead to water dispersion and large-scale outbreaks (Appendix). Cyst infection appears to affect fewer persons in intrafamilial or party-restricted outbreaks (*48*,*49*).

Public health prevention efforts for toxoplasmosis frequently focus on congenital infections to reduce the risk of miscarriage in pregnant women. However, with the occurrence of outbreaks in immunocompetent humans, we suggest that infection control and health education also should be directed to the rest of the population. According to World Health Organization estimates, toxoplasmosis is among the leading foodborne parasitic diseases and in recent years has affected >10.3 million persons worldwide (*50*). Because toxoplasmosis is not a notifiable disease in most countries, reports of toxoplasmosis outbreaks in the literature are needed to increase our understanding of transmission and help reduce the number of outbreaks.

Through our review of published data, we believe the epidemiology of reported toxoplasmosis outbreaks has changed over the past 20 years. Consequently, we suggest that greater attention be paid to the production and disinfection of vegetables, to the quality of drinking and irrigation water, and to the adoption of legislation for tracking outbreaks with the aim of eliminating transmission routes, avoiding exposure, or inactivating the parasite before consumption.

Appendix Additional information on patterns of transmission and sources of infection in outbreaks of human toxoplasmosis around the world.
