# Supplementary material for: Patterns of Transmission and Sources of Infection in Outbreaks of Human Toxoplasmosis
Source: Emerg Infect Dis. 2019 Dec;25(12):2177–82. doi: 10.3201/eid2512.181565 (PMC6874273; doi:10.3201/eid2512.181565)
Supplement: Appendix — Additional information on patterns of transmission and sources of infection in outbreaks of human toxoplasmosis around the world. [file 18-1565-Techapp-s1.pdf]

Article DOI: https://doi.org/10.3201/eid2512.181565

Patterns of Transmission and Sources of Infection in Outbreaks of Human Toxoplasmosis

Appendix

Appendix Table. Complete list of articles included in the systematic review of published data on human toxoplasmosis outbreaks, 1960–March 2018

Ref. no.	Year	Cases, no.	Transmission medium	Parasitic form of *Toxoplasma gondii*	Country	Affected group
1	1965	9	Beef*	Cyst	Brazil	Seminar attendees
2	1966	99	Beef	Cyst	Brazil	University students and faculty
3	1968	5	Beef	Cyst	United States	Diners at snack bar
4	1975	6	Raw lamb	Cyst	United States	Intrafamily
5	1976	3	Raw lamb	Cyst	England	Intrafamily
6	1976	21	Pork	Cyst	Brazil	Intrafamily
7	1979	4	Raw lamb	Cyst	Australia	Intrafamily
8	1980	3	Venison	Cyst	United States	Hunters
9	1987	22	Raw reindeer	Cyst	Canada	Pregnant women
10	1993	17	Raw lamb	Cyst	Brazil	Party participants
11	1994	3	Wild pig viscera	Cyst	South Korea	Merchants
11	1994	5	Pig viscera	Cyst	South Korea	Soldiers
12	1995	16	Game meat	Cyst	French Guiana	Hospitalized patients
13	2005	10	Pork sausage	Cyst	Brazil	Intrafamily
14	2006	6	Beef	Cyst	Brazil	Party participants
15	2006	61	Raw lamb	Cyst	Brazil	Party participants
16	1978	10	Goat milk	Tachyzoite	United States	Intrafamily
17	1983	3	Goat milk	Tachyzoite	Brazil	Intrafamily
18	1988	2	Goat milk	Tachyzoite	Scotland	Intrafamily
19	1976	10	Sand or soil	Oocyst	United States	Intrafamily
20	1977	37	Sand or soil	Oocyst	United States	Horseback riders
21	1979	35	Water	Oocyst	Panama	Soldiers
22	1982	9	Sand or soil	Oocyst	United States	Intrafamily
23	1990	4	Sand or soil	Oocyst	Iraq	Intrafamily
24	1995	110	Water	Oocyst	Canada	Municipality residents
25	2001	426	Water	Oocyst	Brazil	Municipality residents
26	2002	171	Sand or soil	Oocyst	Turkey	Boarding school students
27	2003	11	Water	Oocyst	Suriname	Community neighbors
28	2004	248	Water	Oocyst	India	Municipality residents
29	2004	213	Water	Oocyst	India	Municipality residents
30	2004	40	Sand or soil	Oocyst	Brazil	Municipality residents
31	2008	18	Water	Oocyst	Colombia	Soldiers
32	2009	11	Raw vegetables	Oocyst	Brazil	Industrial restaurant employees
33	2013	73	Açaí	Oocyst	Brazil	Municipality residents

*Transmission route deduced through the epidemiologic evaluation of the article; at the time, the transmission chain of toxoplasmosis had not yet been fully elucidated and the authors suspected ticks and dogs as sources of infection.

References

1. Magaldi C, Elkis H, Pattoli D, de Queiróz JC, Coscina AL, Ferreira JM. Outbreak of toxoplasmosis in a Paulist seminary in Braganza (São Paulo state). Clinical, serological and epidemiological aspects [in Portuguese]. Rev Saude Publica. 1967;1:141–71. PubMed https://doi.org/10.1590/S0034-89101967000200003

2. Magaldi C, Elkis H, Pattoli D, Coscina AL. Epidemic of toxoplasmosis at a university in Sao José dos campos, SP, Brazil [in Portuguese]. Revista Latinoamericana de Microbiologia. 1969;11(1):05–13. PubMed

3. Kean BH, Kimball AC, Christenson WN. An epidemic of acute toxoplasmosis. JAMA. 1969;208:1002–4. PubMed https://doi.org/10.1001/jama.1969.03160060072008

4. Masur H, Jones TC, Lempert JA, Cherubini TD. Outbreak of toxoplasmosis in a family and documentation of acquired retinochoroiditis. Am J Med. 1978;64:396–402. PubMed https://doi.org/10.1016/0002-9343(78)90218-8

5. Fertig A, Selwyn S, Tibble MJ. Tetracycline treatment in a food-borne outbreak of toxoplasmosis. BMJ. 1977;1:1064. PubMed https://doi.org/10.1136/bmj.1.6068.1064

6. Coutinho SG, Morgado A, Wagner M, Lobo R, Sutmoller F. Outbreak of human toxoplasmosis in a rural area. A three year serologic follow-up study. Mem Inst Oswaldo Cruz. 1982;77:29–36. PubMed https://doi.org/10.1590/S0074-02761982000100004

7. De Silva LM, Mulcahy DL, Kamath KR. A family outbreak of toxoplasmosis: a serendipitous finding. J Infect. 1984;8:163–7. PubMed https://doi.org/10.1016/S0163-4453(84)92617-3

8. Sacks JJ, Delgado DG, Lobel HO, Parker RL. Toxoplasmosis infection associated with eating undercooked venison. Am J Epidemiol. 1983;118:832–8. PubMed https://doi.org/10.1093/oxfordjournals.aje.a113701

9. McDonald JC, Gyorkos TW, Alberton B, MacLean JD, Richer G, Juranek D. An outbreak of toxoplasmosis in pregnant women in northern Québec. J Infect Dis. 1990;161:769–74. PubMed https://doi.org/10.1093/infdis/161.4.769

10. Bonametti AM, Passos JN, da Silva EM, Bortoliero AL. Outbreak of acute toxoplasmosis transmitted thru the ingestion of ovine raw meat [in Portuguese]. Rev Soc Bras Med Trop. 1997;30:21–5. PubMed https://doi.org/10.1590/S0037-86821997000100005

11. Choi WY, Nam HW, Kwak NH, Huh W, Kim YR, Kang MW, et al. Foodborne outbreaks of human toxoplasmosis. J Infect Dis. 1997;175:1280–2. PubMed https://doi.org/10.1086/593702

12. Carme B, Bissuel F, Ajzenberg D, Bouyne R, Aznar C, Demar M, et al. Severe acquired toxoplasmosis in immunocompetent adult patients in French Guiana. J Clin Microbiol. 2002;40:4037–44. PubMed https://doi.org/10.1128/JCM.40.11.4037-4044.2002

13. Almeida MABA, Alencar Júnio LR, Carmo GMI, Araújo WNA, Garcia MHO, Reis AK V, et al. Intra-family outbreak of toxoplasmosis, Santa Vitória do Palmar-RS, July 2005 [in Portuguese]. Boletim eletrônico epidemiológico da secretária de vigilância em saúde. 2006;18:1–7.

14. Eduardo MB de P, Katsuya EM, Ramos SRTS, Pavanello EI, Paiva OR, Brito S do N, et al. Investigation of the outbreak of toxoplasmosis associated with the consumption of raw meat dish ("steak tartar"), in the municipalities of São Paulo and Guarujá, SP–November 2006 [in Portuguese]. Boletim Epidemiológico Paulista. 2007;4:1–6.

15. Renoiner EIM, Siqueira AA, Garcia MH, Alves RM, Cardoso ME, Ferreira ABPL, et al. Outbreak of acquired toxoplasmosis, Anápolis-GO, February 2006 [in Porutguese]. Boletim eletrônico epidemiológico da secretária de vigilância em saúde. 2007;(8):1–6. https://portalarquivos2.saude.gov.br/images/pdf/2014/julho/16/Ano07-n08-toxoplasmose-adquirida-go-completo.pdf

16. Sacks JJ, Roberto RR, Brooks NF. Toxoplasmosis infection associated with raw goat's milk. JAMA. 1982;248:1728–32. PubMed https://doi.org/10.1001/jama.1982.03330140038029

17. Chiari CA, Neves DP; Chiari & Neves. Human toxoplasmosis acquired by ingestion of goat's milk [in Portuguese]. Mem Inst Oswaldo Cruz. 1984;79:337–40. PubMed https://doi.org/10.1590/S0074-02761984000300007

18. Skinner LJ, Timperley AC, Wightman D, Chatterton JM, Ho-Yen DO. Simultaneous diagnosis of toxoplasmosis in goats and goatowner's family. Scand J Infect Dis. 1990;22:359–61. PubMed https://doi.org/10.3109/00365549009027060

19. Stagno S, Dykes AC, Amos CS, Head RA, Juranek DD, Walls K. An outbreak of toxoplasmosis linked to cats. Pediatrics. 1980;65:706–12. PubMed

20. Teutsch SM, Juranek DD, Sulzer A, Dubey JP, Sikes RK. Epidemic toxoplasmosis associated with infected cats. N Engl J Med. 1979;300:695–9. PubMed https://doi.org/10.1056/NEJM197903293001302

21. Benenson MW, Takafuji ET, Lemon SM, Greenup RL, Sulzer AJ. Oocyst-transmitted toxoplasmosis associated with ingestion of contaminated water. N Engl J Med. 1982;307:666–9. PubMed https://doi.org/10.1056/NEJM198209093071107

22. Shenep JL, Barenkamp SJ, Brammeier SA, Gardner TD. An outbreak of toxoplasmosis on an Illinois farm. Pediatr Infect Dis. 1984;3:518–22. PubMed https://doi.org/10.1097/00006454-198411000-00007

23. Abood AA. A small outbreak of spontaneous abortion in four patients from two households. Infect Control Hosp Epidemiol. 1992;13:451. PubMed https://doi.org/10.1086/646569

24. Bell A, Gill R, Isaac-Renton J, King A, Martinez L, Roscoe D, et al. Outbreak of toxoplasmosis associated with municipal drinking water—British Columbia. Can Commun Dis Rep. 1995;21–18:161–164. PubMed

25. de Moura L, Bahia-Oliveira LMG, Wada MY, Jones JL, Tuboi SH, Carmo EH, et al. Waterborne toxoplasmosis, Brazil, from field to gene. Emerg Infect Dis. 2006;12:326–9. PubMed https://doi.org/10.3201/eid1202.041115

26. Doganci L, Tanyuksel M, Araz ER, Besirbellioglu BA, Erdem U, Ozoguz CA, et al. A probable outbreak of toxoplasmosis among boarding school students in Turkey. Clin Microbiol Infect. 2006;12:672–4. PubMed https://doi.org/10.1111/j.1469-0691.2006.01449.x

27. Demar M, Ajzenberg D, Maubon D, Djossou F, Panchoe D, Punwasi W, et al. Fatal outbreak of human toxoplasmosis along the Maroni River: epidemiological, clinical, and parasitological aspects. Clin Infect Dis. 2007;45:e88–95. PubMed https://doi.org/10.1086/521246

28. Balasundaram MB, Andavar R, Palaniswamy M, Venkatapathy N. Outbreak of acquired ocular toxoplasmosis involving 248 patients. Arch Ophthalmol. 2010;128:28–32. PubMed https://doi.org/10.1001/archophthalmol.2009.354

29. Palanisamy M, Madhavan B, Balasundaram MB, Andavar R, Venkatapathy N. Outbreak of ocular toxoplasmosis in Coimbatore, India. Indian J Ophthalmol. 2006;54:129–31. PubMed https://doi.org/10.4103/0301-4738.25839

30. do Carmo EL, Póvoa MM, Monteiro NS, Marinho RR, Nascimento JM, Freitas SN, et al. Outbreak of human toxoplasmosis in the District of Monte Dourado, Municipality of Almeirim, Pará State, Brazil [in Portuguese]. Rev Pan-Amazônica Saúde. 2010;1:61–6. https://doi.org/10.5123/S2176-62232010000100009

31. Pino LE, Salinas JE, López MC. Description of an epidemic outbreak of acute toxoplasmosis in immunocompetent patients from Colombian Armed Forces during jungle operations [in Portuguese]. Infectio. 2009;13:83–91. https://doi.org/10.1016/S0123-9392(09)70729-5

32. Ekman CC, Chiossi MF, Meireles LR, Andrade Júnior HF, Figueiredo WM, Marciano MA, et al. Case-control study of an outbreak of acute toxoplasmosis in an industrial plant in the state of São Paulo, Brazil. Rev Inst Med Trop São Paulo. 2012;54:239–44. PubMed https://doi.org/10.1590/S0036-46652012000500001

33. Morais RAPB, Freire ABC, Barbosa DRL, Silva LCT, Pinheiro AF, Costa SS, et al. Acute toxoplasmosis outbreak in the Municipality of Ponta de Pedras, Marajó archipelago, Pará State, Brazil: clinical, laboratory, and epidemiological features [in Portuguese]. Revista Pan-Amazônica de Saúde. 2016;7(esp):143–52. https://doi.org/10.5123/S2176-62232016000500016
